# The emotions of Chinese netizens toward the opening-up policies for COVID-19: panic, trust, and acceptance

**DOI:** 10.3389/fpubh.2024.1489006

**Published:** 2025-01-10

**Authors:** Qiong Dang, Yifei Li, Suping Chen

**Affiliations:** School of Journalism and Communication, Guangxi University, Nanning, China

**Keywords:** Sina Weibo, topic modeling, COVID-19, opening-up policies, public emotions

## Abstract

With the development of social media platforms such as Weibo, they have provided a broad platform for the expression of public sentiments during the pandemic. This study aims to explore the emotional attitudes of Chinese netizens toward the COVID-19 opening-up policies and their related thematic characteristics. Using Python, 145,851 texts were collected from the Weibo platform. Sentiment analysis and topic modeling techniques were employed to reveal the distribution of public emotions and key themes. The study found that the proportions of emotions were as follows: Good (46%), Happy (11%), Anger (17%), Disgust (6%), Sadness (10%), Surprise (2%), and Fear (8%). Through topic analysis, the following main themes were identified: medical resource shortages, healthcare workers, national policies, and COVID-19 sequelae. Based on the results of sentiment and topic analysis, public emotions toward the COVID-19 opening-up policies were categorized into three dimensions: panic, trust, and acceptance. Panic was primarily associated with medical resource shortages, concerns about COVID-19 sequelae, and doubts about policy transparency and fairness. Trust was reflected in public gratitude toward healthcare workers and support for national policies. Acceptance represented the public’s optimism about returning to normal life. The findings demonstrate that changes in public emotions not only reflect the social impact of policy implementation but also highlight the critical roles of medical resource allocation, information transparency, and psychological health support in adjusting pandemic policies. This study provides empirical evidence and theoretical support for the government to develop more precise pandemic control strategies.

## 1 Introduction

Since the outbreak of COVID-19, the Chinese government has implemented a series of strict epidemic prevention and control policies, including city lockdowns, universal nucleic acid testing, centralized quarantine, and health code tracking (1, 2). This strategy, known as “Dynamic COVID-zero,” significantly reduced the spread of the virus by quickly cutting off the transmission chain in the early stages of the epidemic, playing an important role in protecting public health. This strict prevention and control strategy not only demonstrates the government’s organizational capabilities, but also boosts the public’s confidence in fighting the epidemic. However, as the pandemic becomes protracted and globalized, the social, economic, and psychological costs of prevention and control are gradually emerging. The extensive lockdowns have sparked debates among various sectors of society regarding the restrictions they impose on economic activities and personal freedoms. Furthermore, the international community’s widespread shift toward “coexistence with the virus” has further exacerbated the anxiety and dissatisfaction of the Chinese public toward the prolonged lockdown policies.

Against this backdrop, the Joint Prevention and Control Mechanism of the State Council issued the “Notice on Further Optimizing the Implementation of COVID-19 Prevention and Control Measures” (referred to as the “New Ten Guidelines”) on December 7, 2022 (3). This shift marks a significant transformation in prevention and control strategies, moving from “Dynamic COVID-zero “to “categorized management,” and also brings about a greater diversity in public sentiment and discourse. This policy shift directly affects the public’s expectations and trust in the effectiveness of the policy, and also involves a trade-off between individual health safety and socio-economic recovery. Therefore, the issuance of the COVID-19 opening-up policies have sparked widespread emotional reactions and public opinion discussions across various sectors of society, especially on social media.

Social media platforms, as amplifiers of public opinion (4), have become crucial spaces for the public to discuss epidemic policies and express emotions. Globally, social media serves not only as one of the primary channels for obtaining information on the pandemic but also as a key venue for the public to critique policies and express their emotions (5). Chinese social media users share and discuss their views on the COVID-19 pandemic’s opening-up policies in real-time through social platforms, forming a complex emotional ecology. These emotional expressions often reflect the public’s reaction patterns to policy changes and influence broader social public opinion through the platform’s interactive mechanisms. For instance, the spread of positive emotions may enhance public acceptance of policies, while the accumulation of negative emotions may trigger policy disputes or social confrontation (6). Research on these emotional expressions and their dissemination characteristics can not only reveal the psychological state of the public during the policy transition period, but also provide important references for policymakers. Nonetheless, existing research on public sentiment toward COVID-19 opening-up policies remains limited, especially in the context of complex policy changes. How to systematically analyze netizens’ emotional tendencies and the underlying discussion topics still needs further exploration.

Although a large number of studies have been conducted on the public’s attitudes and emotions toward prevention and control policies since the outbreak, most of these studies have focused on the impact of lockdown measures in the early stages of the pandemic, with little attention paid to emotional changes after the policies were relaxed. Existing research has primarily focused on the public’s emotional reactions to strict prevention and control measures during the early stages of the pandemic, such as panic, anger, and anxiety toward lockdown policies (7). However, there has been limited in-depth exploration of the changes and diverse reactions in public sentiment following the implementation of opening-up policies. With the gradual relaxation of policies, the public may experience more complex emotions, such as expectations for policy effects, concerns about public health, and doubts about policy shifts. These dynamic changes remain to be explored in depth. Furthermore, although some studies have mentioned the impact of public sentiment on policy discussions (8–10), they are mostly limited to simple sentiment classification (such as positive or negative sentiment), failing to reveal the association between specific discussion topics and emotional expression. The implementation of COVID-19 pandemic opening-up policies may involve diverse discussion topics, and the complex interactive relationship between these topics and public sentiment needs further exploration. Systematic research on public sentiment toward opening policies and its related topics is still lacking.

Since the outbreak of COVID-19, Weibo, as one of China’s major social media platforms, has hosted a significant volume of public discussions and emotional expressions related to pandemic policies. Emotional expressions by users on Weibo not only influence the dissemination of content but also rapidly impact other users, sometimes triggering viral topics within a short time, particularly on issues concerning personal safety, social fairness, and gender conflicts (11, 12). In the context of a public health emergency, leveraging the vast data from social media to track and analyze the evolution of public sentiment is instrumental. It helps identify public emotions, facilitate psychological intervention, monitor the trajectory of events, and improve the effectiveness of emergency management.

Therefore, this study focuses on the emotional tendencies and related themes of Chinese netizens on the Sina Weibo platform concerning the COVID-19 opening-up policies, aiming to answer the following research questions: RQ1: What are the emotional inclinations of Chinese netizens toward the COVID-19 opening-up policies? RQ2: What are the key themes and discussion topics associated with these emotional inclinations regarding the COVID-19 opening-up policies?

The remainder of this paper is organized as follows. First, we briefly reviewed the background and significance of China’s transition from the “dynamic zero-COVID” policy to the “opening-up” policy during the pandemic. Next, we discussed the role of emotions in the public acceptance of policies and their impact on public opinion guidance, focusing on the characteristics of social media platforms like Weibo as key arenas for public emotional expression and dissemination. Subsequently, we introduced the research methodology, including the process of collecting and analyzing Weibo content, as well as the application of sentiment classification and topic modeling techniques. Through a systematic analysis of Weibo data related to COVID-19 opening-up policies, we presented three main types of public emotions in the findings and discussion section: panic, trust, and acceptance, and uncovered the differences and driving factors behind these emotions. Finally, the study demonstrates that changes in public emotions not only reflect the social impact of policy implementation but also highlight the critical roles of medical resource allocation, information transparency, and psychological health support in the adjustment of pandemic policies.

## 2 Literature review

This section provides theoretical support for understanding the dynamics of public emotions by reviewing the characteristics and types of social media in the context of public emergencies, the relationship between public opinion themes and emotions, and the relevant literature on emotional studies related to COVID-19 in China.

### 2.1 Characteristics and types of social media during public emergencies

In the era of the internet, an increasing amount of information is disseminated through social media. Unlike traditional communication, which separates rationality and emotion into public and private domains, social media has blurred the boundaries between public and private spheres, leading to emotion replacing rationality as the focal point of public sentiment (13). In emergency situations, the public increasingly relies on social media to obtain and share various types of information (8). Users’ emotions and feelings also accumulate and evolve continuously as event information is disclosed and disseminated. In the context of emergencies, social media users exhibit several typical characteristics in their emotional expression. Firstly, emotional variability. Under emergency situations, social media users’ emotions are deeply influenced by information related to events of different natures. As diverse information continues to be disclosed, public emotions are also fluctuating and evolving (9). Secondly, negative emotions are more easily transmitted. Due to the strong negative attributes of sudden public events, they are more likely to trigger negative emotions such as fear, sadness, anxiety, and anger among netizens (10). Thirdly, emotional polarization. Information about emergencies often cannot be clearly manifested in a short period of time, and various rumors are easily spread. These one-sided and false information are highly likely to drive users to adopt extreme attitudes and emotions (14).

From a typological perspective, research on emotions in the context of sudden public events primarily focuses on emotions such as anger and panic. Anger is a tense and unpleasant emotion triggered when wishes are not fulfilled or actions are thwarted (15). In emergency situations, anger is prone to escalate on social media. This is because sudden public events inherently constitute a non-expected behavior that goes against individual expectations and goals. Furthermore, in terms of its spread, anger has been found to be more contagious among users than other emotions (16). Panic is a stress response that people experience when faced with sudden crises (17). Under the stimulus of emergencies, panic is mainly due to people’s uncertain or vague understanding of the danger and controllability of the events they face, which in turn leads to psychological discomfort during the response process (18). Group panic poses great challenges to emergency rescue and post-event handling work.

### 2.2 Thematic and emotional correlation of social media public opinion under sudden public events

Online sentiment does not arise out of thin air, but rather has a profound social background (19). In major sudden public events, there are often diverse topics, which are a concentrated reflection of the public’s attention to the emergency and form the basis for the formation of online public opinion. During the development of emergencies, the content and intensity of topics are also constantly changing (20), and the emotions triggered by different events/topics vary greatly in nature and intensity. In social media, the short-term emotional fluctuations of netizens are often closely related to the trend of a certain topic in unexpected events (21). Therefore, to explain emotional changes, it is crucial to conduct topic mining from massive data, pay attention to different topics and their association with specific emotions, and further explore the patterns of public opinion topics and emotional evolution (22). Conversely, without considering the evolving topic dimension, it is impossible to effectively explain and monitor the types of fluctuating emotions.

### 2.3 Sentiment analysis of Weibo responses to the COVID-19 pandemic in China

During the COVID-19 pandemic, Weibo, as one of China’s largest social media platforms, provided a rich source of data for studying public sentiment dynamics. By analyzing the emotional expressions of Weibo users during different stages of the pandemic, researchers revealed the changing trends of public psychology and their response patterns to policies. In the early stages of the pandemic, fear, anxiety, and anger became the main tone of public sentiment, which was closely related to the uncertainty of the epidemic’s spread, the strain on medical resources, and the chaos of information dissemination (23). However, with the gradual implementation of epidemic prevention and control measures and the enhancement of information transparency, the public’s sentiment has gradually shifted toward hope and optimism (24). This emotional shift not only reflects society’s adaptability to the pandemic but also underscores the importance of government and social resource management (25).

Previous studies have shown that lockdown and quarantine policies have a particularly significant impact on public emotions. In the early stages of the pandemic, these measures led to a surge in negative emotions, such as loneliness, helplessness, and anger toward the restriction of freedom (26). However, these emotions are not static. As lockdown measures continue, the public records their daily lives and shares their quarantine experiences through Sina Weibo to cope with psychological pressure. This online interaction becomes an important means to alleviate negative emotions (27, 28). In addition, research has found that prolonged lockdown policies not only trigger the public’s demand for social support but also promote emotional connections between communities, further strengthening social resilience (29).

Furthermore, as an open platform for information, Weibo not only accelerates the dissemination of epidemic-related information but also provides a pathway for the spread of rumors. Research indicates that epidemic-related rumors often trigger negative emotions such as fear, anger, and distrust, significantly affecting public mental health (30). However, authoritative institutions and media can effectively alleviate these negative emotions by promptly releasing information to refute rumors, thereby enhancing public trust in official information. Additionally, users’ spontaneous refutation of rumors and community discussions on the Weibo platform also play a certain role in suppressing the spread of rumors (31).

Previous studies have shown that sentiment analysis on Weibo primarily relies on natural language processing techniques, including sentiment lexicons, machine learning, and deep learning models (32–34). Sentiment analysis combined with topic modeling can reveal the core issues of public concern and their emotional changes, such as the implementation of epidemic prevention policies, the allocation of medical resources, and the progress of vaccine research and development (35). In addition, the application of artificial intelligence technology has significantly improved the accuracy and timeliness of sentiment analysis (25).

Although existing research has made significant progress in revealing the emotional dynamics of Weibo users during the COVID-19 pandemic, research on the changes in public sentiment after the implementation of COVID-19 opening-up policies remains insufficient. As countries gradually ease their epidemic prevention measures, the emotional expressions and topic concerns of Chinese netizens exhibit complex and diverse characteristics. In-depth exploration of these dynamics is of great significance for understanding social psychological changes and optimizing policy implementation. Overall, although the emotional research conducted during the early stages of the pandemic provided valuable insights for this study, the complexity of public emotions in the context of COVID-19 opening-up policies still need further exploration. This can not only fill the existing research gaps but also assist the government and society in better addressing the psychological and social challenges during the transition phase of the pandemic.

## 3 Materials and methods

### 3.1 Data collection

This study utilizes the API interface provided by the official Weibo open platform^1^ and employs Python to automate data scraping. During the data extraction process, we strictly adhered to the requirements of API Developer Agreement (36) of the Sina Weibo Open Platform, ensuring the legitimacy and compliance of the data source. This agreement is a legal document that developers must adhere to when utilizing the services offered by the Weibo Open Platform. It meticulously outlines the rights and obligations of developers, the rights and obligations of Weimeng Company, intellectual property clauses, privacy-related clauses, conditions for service suspension or termination, and other related content. Our scraping approach was designed to respect user privacy and minimize server impact. No private or sensitive user information was collected, and all data used were publicly accessible.

Specifically, the current study used Python 3.7.0 to collect data related to COVID-19 opening-up policy on the Sina Weibo platform. The keywords used for searching relevant Weibo texts included “pneumonia,” “COVID-19,” “pandemic,” as well as specific topics such as “#20COVIDMeasures,” “#NewTenGuidelines,” “#TravelCardTermination,” “#HealthCodeCancellation,” and “#PandemicRelaxation.”

Data collection lasted for approximately three and a half months from November 21, 2022 to February 5, 2023, during which a total of 145,851 Weibo posts regarding the COVID-19 opening-up policy were retrieved. Subsequently, the collected data was rigorously cleaned to ensure data quality. This cleaning mainly involved removing URLs, punctuation marks, special characters, and any posts with less than 10 characters, which were considered invalid text. Finally, a total of 124,398 valid Weibo posts were kept for further analysis.

### 3.2 Data analysis

#### 3.2.1 Sentiment analysis

The research on sentiment analysis has thrived since the beginning of the 20th century, as exemplified by the work of Pang and Lee (37).” Sentiment analysis is an important branch of natural language processing (NLP) and text mining, which aims to automatically identify, extract, and quantify sentiment tendencies and opinions from subjective texts (38). Its goal is to analyze the sentiment orientation of text (such as positive, negative, or neutral sentiment) as well as more fine-grained sentiment categories (such as anger, joy, sadness, etc.). Modern sentiment analysis methods can be mainly divided into three categories: lexicon-based methods (32), machine learning methods (33), and deep learning methods (34). So far, sentiment analysis has been widely used in the fields of policy evaluation, public opinion monitoring, public health, and social media (29–31). These methods have their own characteristics and significant differences in data requirements, computational resources, development complexity, interpretability, and performance (see Table 1).

**Table 1:** Comparison of three sentiment analysis methods.

Method	Data requirement	Computational resource requirement	Development complexity	Interpretability	Performance
Lexicon-Based	No labeled data required	Low	Simple	Strong	Average
Machine Learning	Labeled data required	Medium	Moderate	Moderate	Good
Deep Learning	Large amounts of labeled data	High	High	Weak	Best

This study employs a lexicon-based approach for sentiment analysis, leveraging its suitability for unlabeled data, high interpretability, and low cost. Using tools like Dalian University of Technology Chinese Emotional Ontology Lexicon (DUTIR), this method enables transparent classification and supports further advanced analysis. This study uses the DUTIR lexicon for its reliability and focus on Chinese linguistic characteristics, making it ideal for efficient, multi-category sentiment analysis of short Chinese texts like Weibo, with scalability and high interpretability. Overall, this study aims to evaluate the functionality of a lexicon-based approach as an initial step in an exploratory study. We chose to begin with a lexicon-based method and plan to explore additional approaches, including machine learning methods, in future work. Moreover, as LDA is a supervised machine learning approach, this study indeed incorporates machine learning techniques.

The DUTIR is constructed based on the six basic emotion categories proposed by Ekman (39). The DUTIR is a Chinese ontology resource compiled and annotated by the Information Retrieval Research Laboratory of Dalian University of Technology under the guidance of Professor Lin Hongfei. The DUTIR is not specifically designed for online text but is a general-purpose Chinese sentiment analysis tool applicable to various text types. However, it is widely used in the analysis of Chinese social media, including platforms like Weibo. This is due to its extensive vocabulary coverage and sentiment classification capabilities, which provide a solid foundation for sentiment analysis of social media text.

The dictionary comprises 27,466 words, describing Chinese vocabulary or phrases from multiple perspectives, including information such as part-of-speech types, sentiment categories, sentiment intensity, and sentiment polarity (40). The types of parts of speech in the DUTIR are divided into seven categories, namely noun, verb, adjective, adverb, network word (nw), idiom, and prepositional phrase. At the same time, each word corresponds to a polarity (positive, negative, neutral) under each category of emotion. Specifically, 0 represents neutrality, 1 represents positivity, 2 represents negativity, and 3 represents both positive and negative connotations. This dictionary categorizes emotions into seven major categories (good, happy, anger, sadness, fear, disgust, and surprise) and 21 subcategories. The dictionary categorizes emotional intensity into five levels, namely 1, 3, 5, 7, and 9. Among them, nine signifies the strongest emotional intensity, while 1 denotes the weakest (41).

The specific steps of sentiment analysis are as follows: First, the Pandas library is called to read and clean the data. Next, we use the regular expression module re to clean the raw data and remove invalid content (such as URL links, special characters, HTML tags, etc.). Secondly, import the data into the DUTIR. Thirdly, we utilize the Jieba word segmentation tool to segment each Weibo text and load a customized stop word list, removing meaningless functional vocabulary (Such as “of,” “the,” “and”). Fourthly. we perform sentiment word matching on the word segmentation results of each text and calculate the distribution of various sentiment categories, Lastly, based on the matched sentiment distribution, we calculate the main sentiment category and overall sentiment distribution of each text.

To validate the analytical effectiveness of the DUTIR, this study adopted a dual-researcher independent annotation strategy. Each researcher randomly selected 3,000 Weibo text samples, covering various sentiment orientations and categories. Both researchers conducted independent annotations following a unified standard. The Cohen’s Kappa coefficient for inter-rater reliability exceeded 0.8, indicating that the annotations were highly reliable and suitable as a gold standard.

Subsequently, DUTIR was applied to classify the sentiment of the same samples, and the results were compared with the human-annotated gold standard. The analysis revealed that the lexicon achieved an accuracy of 87%, a recall of 83%, and an F1 score of 0.85, all meeting the “excellent validity” criteria in academic research (accuracy ≥85%, recall ≥80%, F1 score ≥ 0.8). These findings demonstrate that DUTIR possesses high reliability and applicability in sentiment analysis of Weibo texts.

#### 3.2.2 LDA topic analysis

Latent Dirichlet Allocation (LDA) is a widely used topic modeling method, with its theoretical foundation primarily derived from the work of Blei et al. (42). The Latent Dirichlet Allocation (LDA) model is a document topic generation model based on a three-layer Bayesian probabilistic framework. It is primarily trained through unsupervised learning mechanisms, extracting latent topic information from massive text data, thereby revealing the underlying topic structure in large-scale document collections or corpora (22, 42). The main advantages of LDA topic models lie in their ability to provide an efficient and automated method for mining the latent semantic structure of massive text data. They output probability distributions, supporting fine-grained topic analysis and content clustering. These models are suitable for text analysis tasks across multiple domains, demonstrating high flexibility and scalability.

This study conducts LDA topic model analysis on the collected Weibo texts, and employs Python language, gensim, and pyLDAvis libraries for topic generation and visualization of topics. The main steps of LDA topic analysis are as follows: Firstly, we performed data preprocessing on the original text, including removing special characters, word segmentation, removing stop words, and filtering important parts of speech (such as nouns and verbs), to ensure the quality of the corpus. Subsequently, based on the cleaned corpus, we constructed a dictionary and a corpus, which store all individual words and their word frequency distribution, respectively, providing a foundation for model training. In model training, Gensim tool is used to train models with topic numbers ranging from 1 to 15 one by one. Bayesian inference is employed to optimize the assignment of words to topics, generating keyword distributions and weights for each topic. To determine the optimal number of topics, consistency (measuring the semantic coherence of topics) and perplexity (measuring the generative ability of the model) are calculated separately, and their variation trends are plotted. Ultimately, based on the principle of achieving peak consistency and a slow decline in confusion, we chose 15 as the optimal number of topics. Using this topic number model, we extracted the core keywords and their weight distribution for each topic.

## 4 Results

### 4.1 Description of Weibo posts

**Figure 1:**
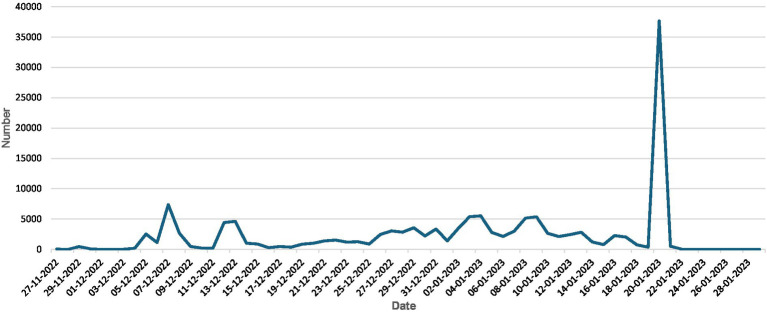
Number of Weibo posts concerning COVID-19 opening-up policies.

Figure 1 presents the dynamic changes in the number of valid texts per day from November 21, 2022, to February 5, 2023. From the overall trend of public discussions on the COVID-19 reopening policies, the volume of discussions experienced a steady increase in the early stages following the policy announcement, a surge during the Spring Festival travel rush (43), a concentrated peak during the Chinese New Year, and a rapid decline after the holiday, showing significant stages.

Major policy changes often trigger public attention and uncertainty (44). Following the release of the “New Ten Guidelines” on December 7, 2022, an initial peak of discussion emerged, reflecting the public’s expectations and concerns regarding the relaxation of policies. The emotional fluctuations during this period align with Wang’s (45) perspective that policy changes can lead to an “emotional arousal effect,” making individuals more inclined to express emotions such as anxiety or anticipation. The start of the Spring Festival travel rush on January 7, 2023 has triggered large-scale personnel movements and also led to a significant increase in discussions on social media regarding the COVID-19 opening-up policies. This phenomenon can be explained through risk perception theory (46).

The peak of the entire period occurred on January 22, 2023, during the Chinese New Year, with a discussion volume of over 37,000 posts. One possible explanation is that the Spring Festival is an important moment in Chinese culture that emphasizes family reunion, but the uniqueness of the 2023 Spring Festival lies in the interweaving of epidemic transmission and festive emotions: the health risks during large-scale gatherings have exacerbated public anxiety (47), especially concerns about infection among the older adults. At the same time, the reunion sentiment during the Spring Festival has also stimulated people’s optimistic attitude toward policy openness. The emotional complexity during this stage aligns with Lazarus and Folkman’s (48) “stress and coping theory”: when faced with threats, people tend to exhibit emotional ambivalence, such as feeling both anxiety and anticipation simultaneously.

After the Spring Festival (after January 22), public discussions on the COVID-19 opening-up policies declined rapidly. This phenomenon can be explained by the issue-attention cycle (49), which suggests that after prolonged and intense discussion, public interest in the topic rapidly declines. After the New Year, as life returned to normal, public attention gradually shifted to other societal or personal matters, leading to a stabilization of public discourse.

The discussion curve of netizens regarding the COVID-19 reopening policies reflects the interplay between policy adjustments, social activities (such as the Spring Festival travel season and Chinese New Year), and public emotions. The emergence of peaks is not only a direct result of policy implementation but also a comprehensive reaction from the public to health risks, the effectiveness of policy execution, and cultural festive emotions.

### 4.2 Identify netizens’ sentiments

**Figure 2:**
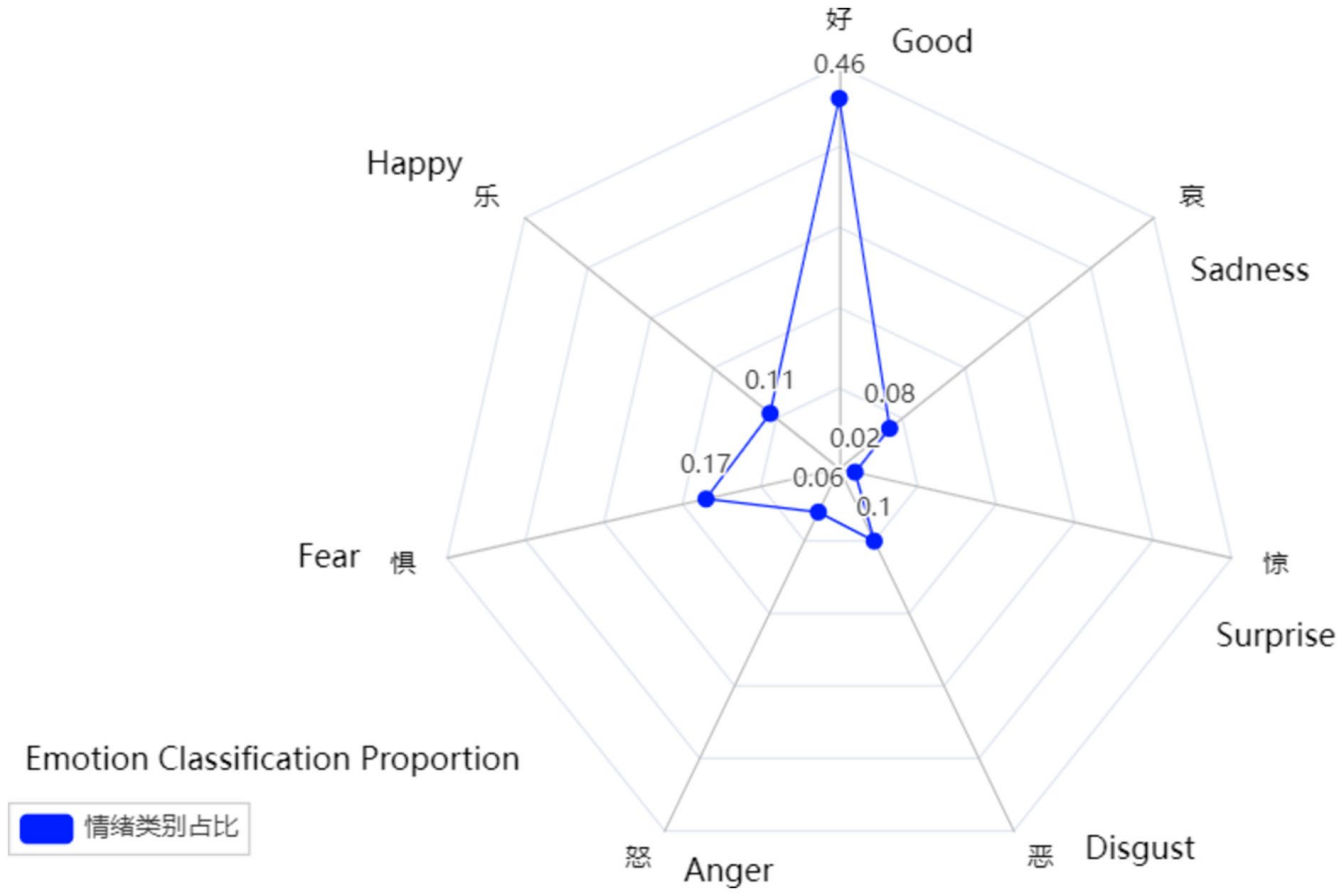
The proportion of each emotion category.

According to the sentiment classification results, the proportions of various emotions are as follows (see Figure 2): Good (46%), Happy (11%), Anger (17%), Disgust (6%), Sadness (10%), Surprise (2%), and Fear (8%). The emotions of “Good” and “Happy” together account for the highest proportion, reaching 57%. This result indicates that the majority of netizens exhibited positive emotional responses in the context of the COVID-19 opening-up policies. This high proportion likely reflects the public’s relatively high acceptance of the scientific basis of the policies and the positive emotional effects brought about by their implementation.

In the sentiment analysis, the two researchers manually categorized the content by considering the context and position of high-frequency keywords (see Table 2) in the Weibo texts. We did not rely solely on word frequency but also carefully examined the surrounding context to ensure accurate sentiment classification. To assess the consistency of the classification, we used Cohen’s Kappa coefficient, which resulted in a value of 0.834, indicating high inter-rater reliability and strong structural validity.

**Table 2:** Top 10 most relevant keywords for each of the seven emotion categories.

Good	Keyword	Expert	Health	Hope	Certain	Thanks	Support	Friend	Cannot	Indeed	Believe
	Frequency	7,162	3,796	2,982	2,517	1,248	1,204	983	943	920	830
	Part of Speech	noun	adj	verb	adv	verb	verb	noun	verb	adv	verb
	Intensity	3	3	5	5	3	5	9	5	5	7
Happy	Keyword	Rise	Peace	Happiness	Haha (Laughter)	Restore	Safety	Resolve	New Year	Normal	Good Things
	Frequency	1,575	1,412	1,191	1,078	991	783	726	684	487	450
	Part of Speech	verb	adj	adj	nw	verb	adj	verb	noun	adj	verb
	Intensity	7	7	5	7	1	3	1	5	3	5
Fear	Keyword	Virus	Harmful	Disaster	Fearful	Cautious	Difficulty	Rescue	Priority	Cases	Panic
	Frequency	2,500	393	389	264	250	188	174	173	163	132
	Part of Speech	noun	adj	adj	adj	verb	noun	verb	noun	noun	adj
	Intensity	5	3	3	3	1	1	1	7	5	7
Sadness	Keyword	Suffering	Impossible	Hospitalized	Sick	Speechless	Pitiable	Painful	Hardship	Funeral Home	Suffering
	Frequency	1,156	458	427	416	364	266	231	221	212	118
	Part of Speech	adj	verb	noun	verb	nw	adj	noun	adj	noun	noun
	Intensity	5	7	7	5	5	5	7	5	5	9
Disgust	Keyword	Not	Useless	Gone	Severe	But	Bad	Anxious	Crazy	Nonsense	Doubtful
	Frequency	4,921	1,033	989	935	783	780	394	314	306	182
	Part of Speech	verb	prep	adj	adj	verb	adj	adj	nw	idiom	verb
	Intensity	5	5	3	3	5	3	3	7	7	9
Anger	Keyword	Outbreak	Complaint	Anger	Deserve	Relied	Punishment	Protest	Shit	Fury	Vent
	Frequency	170	75	61	41	26	24	21	20	20	14
	Part of Speech	verb	verb	noun	adj	nw	verb	verb	nw	adj	adj
	Intensity	7	7	3	3	5	5	5	7	5	5
Surprise	Keyword	Original	Gosh	Strange	Curious	Marvel	Incredible	Cannot Bear	Shocked	Unbelievable	Too Late
	Frequency	332	249	109	70	47	23	23	18	16	15
	Part of Speech	verb	nw	adj	adj	adj	noun	idiom	nw	idiom	idiom
	Intensity	5	7	5	3	5	9	9	7	5	5

From the data on the positive emotion categories “Good” and “Happy,” the high-frequency words within these categories show distinct characteristics in terms of frequency, part of speech, and intensity. The “Good” emotion primarily reflects trust and support for experts, health, and policy implementation. In contrast, the “Happy” emotion predominantly conveys optimism about the return to normalcy, the rebuilding of social connections, and an increased sense of security.

In the “Good” emotion category, high-frequency terms such as “experts” (Frequency = 7,162), “health” (*F* = 3,796), and “hope” (*F* = 2,982) reflect the public’s trust in scientific guidance, emphasis on health, and anticipation for improved living conditions. Verbs like “hope,” “thank,” “support,” and “believe” constitute half of the terms, indicating active public support for COVID-19 opening-up policies. The term “friends” (*F* = 983, intensity 9) highlights the desire to restore social relationships, while the high intensity of “believe” (intensity 7) underscores confidence in policies and expert opinions. Overall, the “Good” emotion data reveal a positive public attitude toward scientific decision-making, health protection, and social recovery.

In the “Happy” emotion category, terms like “rise” (*F* = 1,575), “peace” (F = 1,412), and “happiness” (F = 1,191) demonstrate the public’s longing for normalcy, social stability, and personal well-being. Expressions such as “haha (laughter)” (1,078 occurrences) convey joy and relief as the pandemic subsides. The high intensity of “peace” (intensity 7) emphasizes the importance of a stable social environment in fostering optimism. Verbs like “restore” (*F* = 991) and “resolve” (*F* = 726), though of lower intensity, are frequently used, reflecting the public’s focus on addressing practical issues and restoring social order. These findings illustrate that positive emotions are closely tied to specific societal goals. Overall, these findings highlight the public’s strong anticipation for the resumption of normal life, social security, and a sense of happiness, along with their positive emotions.

The negative emotion category encompasses “Fear,” “Sadness,” “Disgust,” and “Anger,” reflecting public emotions such as fear, sorrow, rejection, and anger in response to the COVID-19 reopening policies. These emotions, characterized by high-frequency words and emotional intensity, reveal the public’s strong concerns about health risks, social inequities, and issues in policy implementation.

In the “Fear” emotion category, high-frequency words reflect public concerns about health risks and insufficient medical support during the pandemic. “Virus” (*F* = 2,500) highlights fear of its persistence, reinforced by “afraid” (*F* = 393) and “panic” (*F* = 132, intensity 7), expressing anxiety over infection risks and policy uncertainty. Verbs like “rescue” (*F* = 174) and “be cautious” (*F* = 250) emphasize emergency care and self-protection, while nouns such as “cases” (*F* = 163) and “priority” (*F* = 173, intensity 7) reveal concerns about worsening conditions and resource allocation. Overall, public fear centers on health threats, infection risks, and insufficient medical resources.

In the “Sadness” emotion category, high-frequency words such as “uncomfortable” (F = 1,156) and “sad” (*F* = 221) reflect widespread sorrow caused by physical and psychological impacts. “Unfortunate” (*F* = 118, intensity 9) highlights grief from loss and economic hardship, amplified by “funeral home” (*F* = 212, intensity 5). Verbs like “sick” (*F* = 416) and “hospitalized” (*F* = 427) express helplessness and urgent medical needs, while adjectives like “speechless” (*F* = 364) and “regrettable” (*F* = 266) convey regret and helplessness over losses and injustices. These words reveal the profound life pressures and sorrow experienced by the public during the pandemic.

In the “Disgust” and “Anger” emotion categories, high-frequency words and emotional intensity reflect public dissatisfaction with policy implementation during the pandemic, particularly regarding information transparency, execution effectiveness, and resource allocation fairness. In “Disgust,” words like “not” (*F* = 4,921), “useless” (*F* = 1,033), and “bad” (*F* = 780) highlight skepticism toward policy effectiveness. “Doubt” (*F* = 182, intensity 9) reveals mistrust in policy transparency and credibility, driven by information asymmetry. Similarly, in “Anger,” words like “complaint” (*F* = 75, intensity 7) and “protest” (*F* = 21, intensity 5) express frustration over insufficient communication and transparency. In terms of execution effectiveness, “Disgust” includes “nonsense” (*F* = 306, intensity 7), showing aversion to misinformation, and “severe” (*F* = 935), reflecting rejection of pandemic challenges and resource inequities. In “Anger,” “outbreak” (*F* = 170, intensity 7) and “punishment” (*F* = 24, intensity 5) convey frustration with worsening conditions and insufficient resource allocation, underlining these issues as key triggers of public emotion.

### 4.3 Identify latent themes

The LDA (Latent Dirichlet Allocation) topic model is a three-layer Bayesian model based on the “document-topic-word” framework (42). It assumes the existence of ***K*** topics within a collection (***K*** being the optimal number of topics), with topics defined as probability distributions over words. The number of topics, ***K***, is a necessary input and is determined using perplexity (50). When perplexity is minimized, the model achieves its best performance for that particular number of topics, thus determining ***K***. The calculation formula for perplexity, commonly used to measure the quality of predictions made by an LDA topic model, is as follows (42):


(1) $$\mathrm{Perplexity}\;\left(D\right)=\exp.\left\{-\frac{\sum_{d=1}^{M}\log_{P}\left(w_{d}\right)}{\sum_{d=1}^{M}N_{d}}\right\}$$

D: Represents the test document set; M: Refers to the total number of documents; $p\left(w_{d}\right)$ : Denotes the probability of generating document *d* as computed by the model; $N_{d}$ : Indicates the number of words in document.

**Figure 3:**
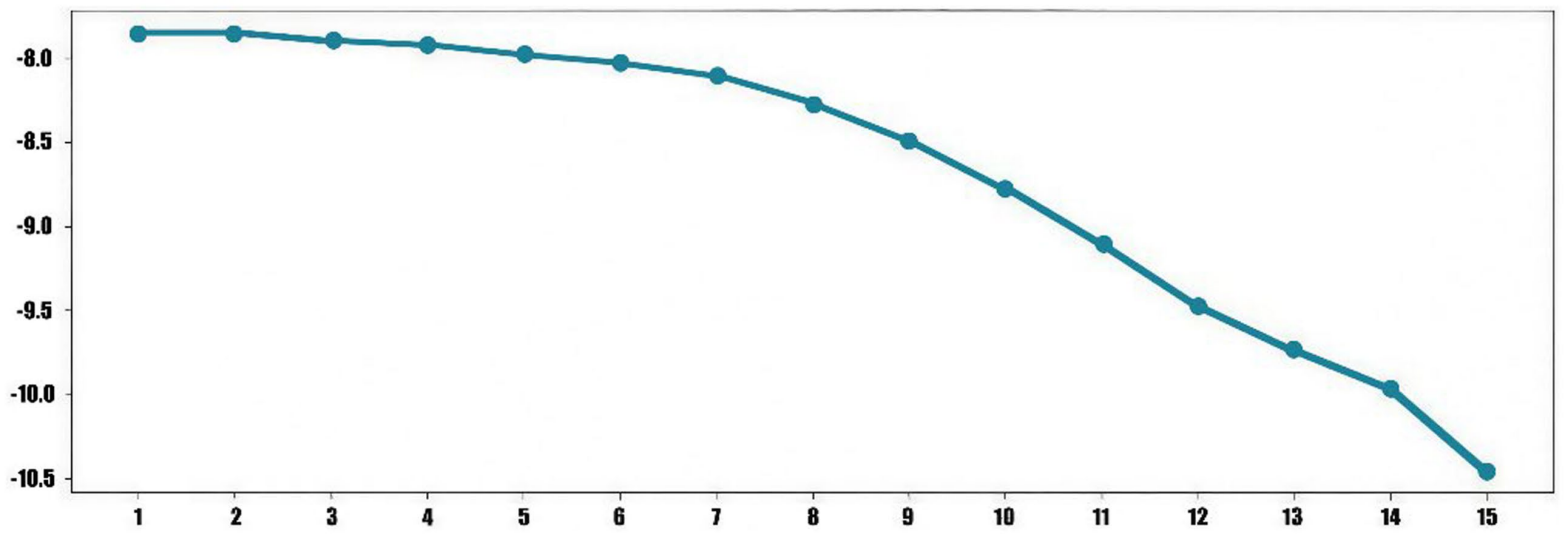
Perplexity. The horizontal axis represents the number of topics, and the vertical axis represents the perplexity value.

**Figure 4:**
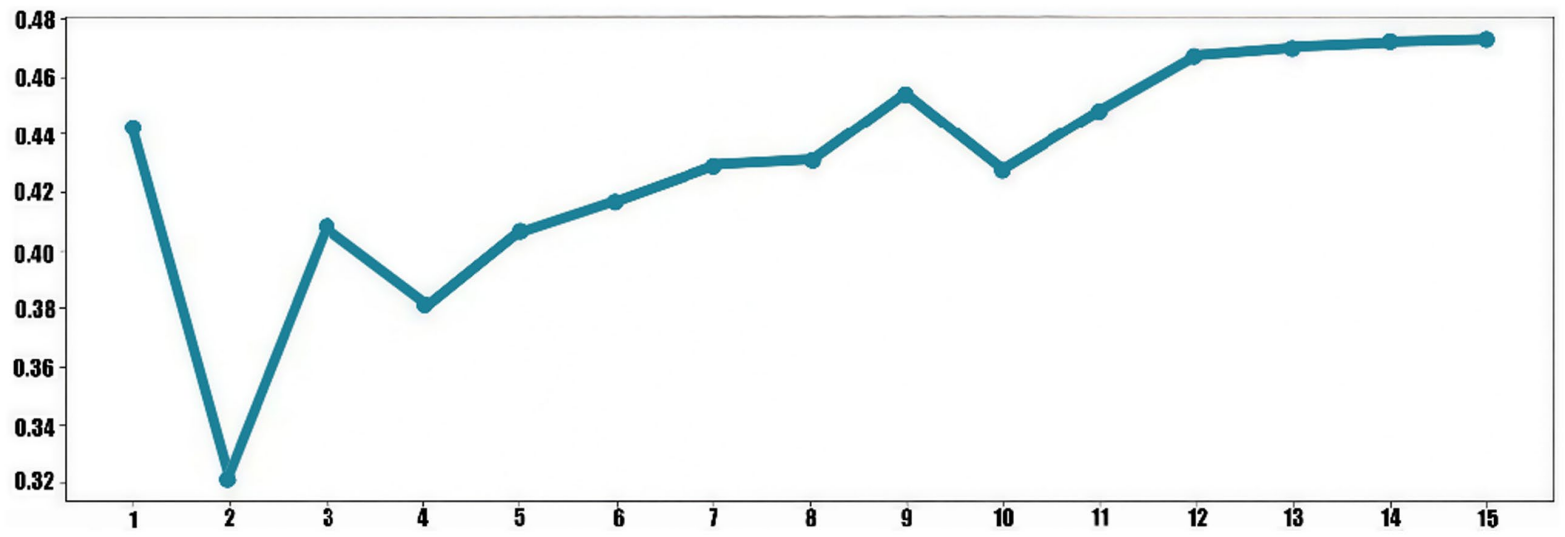
Coherence. The horizontal axis represents the number of topics, and the vertical axis represents the coherence value.

Topic coherence score indicates whether the high-probability words associated with each topic are semantically consistent; the higher the score, the better the model’s performance. In this study, the LDA topic model was evaluated using two metrics: perplexity and coherence, to determine the optimal number of topics. From the perplexity trend graph (see Figure 3), it is evident that the perplexity value reaches a low point (−10.05) when the number of topics is 15, indicating that the model achieves the best fit to the data at this number of topics. Similarly, from the coherence trend graph (see Figure 4), the coherence score reaches a local peak (0.47) when the number of topics is 15. This suggests that at this number of topics, the semantic association among words within each topic is strongest, resulting in higher semantic clarity of the topics. Therefore, selecting 15 topics (see Table 3) ensures high semantic quality of the topics while balancing the model’s complexity and generalization ability, making it the optimal choice for the number of topics.

**Table 3:** Top 10 most relevant keywords for each of the 15 topics.

Topic1	Keywords	Doctor	Mask	Body	First wave	Pharmacy	Price increase	Clinical	Positive (Recovery)	Entire	Minor illness
	Probability	0.132	0.124	0.044	0.028	0.021	0.011	0.011	0.011	0.009	0.008
Topic2	Keywords	Country	Local	Individual	Itinerary	Government	Data	Control	Zhang Wenhong	Dynamic	Plan
	Probability	0.091	0.065	0.064	0.038	0.022	0.015	0.014	0.013	0.012	0.011
Topic3	Keywords	Pneumonia	Doctor	Society	Official	Outpatient	Oxygen	Nurse	Hospital	Antibiotics	Professiona
	Probability	0.058	0.055	0.023	0.020	0.019	0.018	0.014	0.012	0.011	0.009
Topic4	Keywords	Beijing	Family	Pfizer	Antiviral	Medicine	Stay Home	Effective Drug	Crematorium	Mild Symptom	Miracle Drug
	Probability	0.053	0.050	0.042	0.033	0.029	0.028	0.026	0.026	0.019	0.011
Topic5	Keywords	Expert	Focus	Epidemic	Prediction	Impact	Population	Science Popularization	Wuhan	Antibody	Prevention
	Probability	0.301	0.144	0.13	0.047	0.021	0.012	0.01	0.008	0.007	0.005
Topic6	Keywords	Nucleic Acid	Child	Policy	Traditional Medicine	Antigen	Testing	Mask	Health Commission	Western Medicine	Patent Medicine
	Probability	0.185	0.051	0.04	0.032	0.023	0.023	0.021	0.011	0.009	0.007
Topic7	Keywords	Data	Older adults	Virus Strain	Hometown	County	School Opening	Patient	Ventilator	Lung Cleaning	Oxygen Generator
	Probability	0.051	0.039	0.026	0.024	0.018	0.016	0.014	0.013	0.012	0.009
Topic8	Keywords	Budesonide	Medical Care	Antipyretic	Medicine	Resources	Infusion	Lotus	Price	Acetaminophen	Panic
	Probability	0.050	0.045	0.030	0.022	0.020	0.012	0.010	0.010	0.008	0.006
Topic9	Keywords	Virus	China	Home	Older adults	Rural	Measures	Prevention	Mutation	Data	Infection Rate
	Probability	0.097	0.070	0.053	0.046	0.036	0.034	0.016	0.016	0.014	0.013
Topic10	Keywords	Everyone	Economy	Thanks	Work	Sick	Management	Family	Pain	Caution	Medicine
	Probability	0.203	0.041	0.041	0.036	0.030	0.024	0.019	0.019	0.015	0.013
Topic11	Keywords	Older adults	Life	Comments	Care	Young People	Direction	History	Nursing	Meaning	Responsibility
	Probability	0.216	0.054	0.037	0.025	0.024	0.021	0.020	0.018	0.017	0.006
Topic12	Keywords	COVID-19	Severe	Basic	Patient	Common	Flu	Medication	Disease	Hospitalization	Life-saving
	Probability	0.125	0.066	0.036	0.021	0.016	0.016	0.015	0.013	0.013	0.011
Topic13	Keywords	Ordinary people	Summit	Blogger	Nurse	I tested positive for COVID-19	West Medicine	Cough	Ministry of Foreign Affairs	Physician	Wen Hong
	Probability	0.069	0.054	0.028	0.026	0.014	0.014	0.013	0.011	0.009	0.007
Topic14	Keywords	Hospital	Senses	Vaccine	Lianhua Qing	Symptoms	Positive	Mortuary	All tested positive	Hospital beds	Temporary hospital
	Probability	0.131	0.073	0.060	0.059	0.046	0.016	0.014	0.012	0.010	0.009
Topic15	Keywords	Suggestion	People	Prevention	Nationwide	Probability	Life	Side effects	Cost	Body temperature	Sequelae
	Probability	0.098	0.055	0.035	0.029	0.022	0.021	0.019	0.012	0.010	0.010

Topic strength refers to the relative proportion of each topic in the entire corpus after clustering (51). The higher the topic strength, the more frequently the topic appears in the corpus, indicating that the content of this topic contributes more to the overall corpus (42). Table 4 shows that the topic weights are distributed relatively evenly, with the highest, Topic 1, accounting for 9%, and the lowest, Topic 15, accounting for 5.1%. The differences in weight between the topics are minimal, suggesting that each topic contributes to the overall content, and no single topic clearly dominates the entire corpus. Presenting the 15 topics directly would complicate the analysis and make the results difficult to interpret. Therefore, this study aggregates and clusters the 15 topics into 4 higher-level themes through manual categorization, which helps enhance the focus, interpretability, and logical clarity of the analysis.

**Table 4:** Topic intensity.

Topic	1	2	3	4	5	6	7	8
Intensity	9%	8%	7.4%	7.3%	7.1%	7%	6.6%	6.5%
Topic	9	10	11	12	13	14	15	Total
Intensity	6.3%	6.3%	6.2%	6%	5.6%	5.6%	5.1%	100%

Manual inductive topic clustering is a systematic process based on logical reasoning and semantic analysis. Manual induction is the core method for identifying themes and patterns in the analysis. Researchers search for semantic and contextual associations in the data and, in line with the research objectives, aggregate scattered content into clearer themes. Ryan and Bernard (52) explored the combination of manual induction and computer-assisted analysis (such as topic modeling), arguing that manual induction can effectively address the semantic limitations of computational methods. They advocate for researchers to use their experience and background knowledge to inductively refine and adjust the automatically generated topics during the topic identification process.

Therefore, we use manual induction and clustering to consolidate the 15 evenly distributed topics into 4 higher-level themes (see Table 5), which helps enhance the focus, interpretability, and logical clarity of the analysis. Therefore, the two researchers independently classified the topics based on the same dataset and inductive rules. The Cohen’s Kappa coefficient ranges from [−1, 1], with a result between 0.61 and 0.80 indicating good consistency, and between 0.81 and 1 indicating excellent consistency (53). After independently classifying the topics, the Kappa value was 0.84.6, indicating a high level of consistency, which meets the reliability requirements of scientific research.

**Table 5:** The identified four themes.

Theme	Theme label	Topics	Interpretation	Emotional inclination
1	Medical resource shortages	Topics 1, 8, 12, 14	It reflects the social panic and concern triggered by the shortage and unequal distribution of medical resources, involving issues related to medications, medical equipment, and rural healthcare.	Negative
2	Healthcare Workers	Topics 3, 11, 10, 13	The topic reflects the public’s gratitude toward healthcare workers, emphasizing their efforts and contributions during the pandemic. It also highlights the public’s concern for the health of their families and their support for epidemic prevention workers.	Positive
3	National policies	Topics 2,5, 7,9	It focuses on the public’s trust and support for the country’s COVID-19 reopening policies and data transparency.	Positive
4	COVID-19 sequelae	Topics 4, 15, 6	This topic reflects the public’s concerns about the health sequelae, psychological stress, and the high-risk groups affected by the pandemic.	Negative

## 5 Discussion

The relationship between topics and emotions is central to public opinion research, particularly during emergencies where they closely interact (53). Topics represent public discussion content, while emotions reflect attitudes and responses. Topics provide context for emotions, which in turn influence topic spread and perception (54). High emotional intensity amplifies topic dissemination and shapes public cognition. For example, trust and gratitude often focus on healthcare workers or successful policies, reinforcing support for positive topics (55). Based on thematic and sentiment analyses, this study identifies three core emotions of Chinese netizens toward the COVID-19 opening-up policy: panic, trust, and acceptance.

### 5.1 Panic: health risks and resource shortages

#### 5.1.1 Panic for medical resource shortages

After the implementation of COVID-19 opening-up policies, public panic over medical resource shortages became a key topic on social media. Weibo data highlights concerns about medication shortages, hospital bed availability, and funeral home strain, reflecting supply–demand contradictions and public anxiety over pandemic management.

In the Weibo data, High-frequency keywords such as “Ibuprofen,” “antipyretics,” and “special medicines” reveal anxiety over medication shortages during the pandemic peak, when soaring infections led to a demand surge and market imbalances. Social media amplified medication hoarding, spreading individual fears into collective discussions (56, 57). Similarly, terms like “hospital beds,” “hospitalization,” and “makeshift hospitals” indicate public skepticism about healthcare system capacity, particularly for high-risk groups like the older adults and children. Verbs like “rescue” and “be cautious” highlight fears over health risks due to insufficient medical resources. As one netizen wrote: *“The shift from the zero-COVID policy to full reopening has exposed us to the immense pressure on the healthcare system. The surge in actual infections has led to a severe strain on medical resources, pushing them to the brink of collapse.”* This resonates with widespread concerns about shortages in medication and hospital beds.

A notable finding is the significant discussion about funeral home strain, rarely mentioned in earlier literature. Upon examining the original Weibo posts, we found that the high-frequency term “funeral home” is often associated with keywords like “queue” and “cremation,” which reflects fears of rising death tolls and inadequate end-of-life services. This “visibility of death” broke Chinese cultural taboos around discussing death (58), intensifying public psychological reactions and criticism of healthcare inadequacies. Keywords such as “complaint,” “outbreak,” and “anger” express dissatisfaction with unequal resource distribution and inefficient policy implementation. Rural areas face acute medication and facility shortages, exacerbating public anxiety (59). Poor coordination in drug distribution and hospital scheduling highlights structural deficiencies in emergency management, eroding trust in government.

Weibo discussions amplified localized issues like medication hoarding and funeral home strain into perceived nationwide crises. Declining trust in the government, coupled with misinformation and exaggerated narratives, further heightened social panic. The rapid dissemination of these emotions, rarely observed in traditional media, reveals a key characteristic of this study.

#### 5.1.2 Concerns about COVID-19 sequelae

Through Weibo data analysis, keywords such as “throat,” “ventilator,” “lung cleansing,” “sequelae,” “illness,” and “hospitalization” emerged as central topics in public discussions about COVID-19 aftereffects. These keywords reflect widespread public concerns about the long-term health impacts of COVID-19 infections. Notably, these terms are also associated with symptoms like “diarrhea,” “abnormal body temperature,” and “throat discomfort,” highlighting the manifestations of aftereffects across multiple bodily systems and their complexity.

COVID-19 sequelae often involve multi-system health issues, with diverse manifestations and varying durations. Studies have shown that COVID-19 sequelae can affect the respiratory system, nervous system, digestive system, and psychological and cognitive functions (60, 61). The appearance of keywords such as “throat” and “lung cleansing” on Weibo reflects public concerns about the long-term effects on the respiratory system, while symptoms like “diarrhea” and “abnormal body temperature” further reveal the complexity of digestive system and systemic issues.

These symptoms align with descriptions of COVID-19 sequelae in the literature. Zeng et al. (62) noted that common symptoms of COVID-19 sequelae include fatigue, cough, shortness of breath, and persistent indigestion. The diversity and long-term nature of these symptoms have raised public doubts about the predictability of post-COVID health recovery, further exacerbating anxiety.

Discussions related to “sequelae” on Weibo also reveal the public’s demand for healthcare system support and expectations for mental health assistance. The long-term nature and multi-system impact of COVID-19 sequelae imply that patients require ongoing medical treatment and psychological interventions. However, the current lack of comprehensive research on the mechanisms of sequelae has resulted in limited public confidence in the effectiveness of treatments.

Studies have shown that the incidence of depression and anxiety has significantly increased among COVID-19 sequelae patients (63). Discussions in Weibo data reveal that, when facing symptoms such as “diarrhea” and “throat discomfort,” the public is not only concerned about physical health issues but also anxious about the impact on social and economic activities. For instance, some patients reported being unable to return to normal life and work due to persistent symptoms, further exacerbating the burden on families and society. As one netizen noted: *“The COVID-19 opening-up policy has caused unprecedented psychological and economic shocks to people*.” This statement highlights the dual impact of the reopening policy on society and individuals, affecting not only public physical health but also having profound effects on mental health and economic conditions.

Following the Covid-19 opening-up policy, the surge in infection numbers and the lack of sufficient scientific understanding of sequelae have heightened the public’s perceived risk to their own health. This risk perception, as expressed on social media, not only demonstrates a high level of concern about health issues but also reflects public skepticism about the healthcare system’s capacity and the details of policy implementation.

#### 5.1.3 Doubts about policy transparency and fairness

Analysis of Weibo data reveals that high-frequency words such as “not,” “doubt,” and “serious” reflect widespread public skepticism regarding the transparency and fairness of the COVID-19 opening-up policies. This skepticism extends not only to the policymaking process but also to the outcomes of policy implementation. Emotional terms like “nonsense” further highlight strong public dissatisfaction with misinformation and inadequate policy communication. A lack of transparency can directly undermine public trust in government (64, 65). The emotional expressions observed in Weibo discussions indicate that public dissatisfaction with the policies is primarily centered on delays in information disclosure, unclear policy justifications, and uncertainties in policy outcomes.

A lack of policy transparency is one of the key reasons behind public skepticism. During the implementation of the COVID-19 opening-up policies, the government failed to promptly disclose the rationale and details of policy formulation and execution, leading the public to question the scientific validity and rationality of these policies. For example, “doubt” and “not” became frequently mentioned keywords on Weibo, reflecting the public’s negative attitude toward policy transparency and credibility. This phenomenon aligns with the perspective of Lee and Lee (66), who argue that information asymmetry exacerbates public distrust and may trigger broader societal emotional fluctuations.

Meanwhile, Weibo data also reveals public criticism of policy fairness. Some users highlighted the unequal distribution of medical resources, particularly in areas with significant urban–rural disparities. The scarcity of medical resources in rural and remote areas has further intensified public sentiments of injustice regarding policy implementation. Fairness is a fundamental ethical principle in public health policy. When inequalities in resource allocation are perceived by the public, trust in the policy tends to decline (67, 68). Previous studies have shown that public trust in the government relies on the competence, fairness, and transparency of its policies. In this study, public distrust in the policies is clearly closely related to poor implementation and a lack of transparency (69, 70). The repeated mentions of “serious” and “nonsense” on Weibo not only express the public’s concerns about issues in policy implementation but also reflect their rejection of the fairness of the policies.

### 5.2 Trust: healthcare workers and national policies

#### 5.2.1 Gratitude toward healthcare workers

Weibo data reveals that high-frequency keywords such as “Zhang Wenhong,” “nurse,” “hospital,” “medical staff,” “gratitude,” and “support” prominently reflect the public’s deep appreciation for the dedication of healthcare workers during the pandemic. The discussions not only demonstrate a high level of recognition for the professionalism and responsibility of healthcare personnel but also indicate an overall increase in societal trust in the healthcare system. Many Weibo users mentioned names like “Zhang Wenhong,” highlighting the public’s strong reliance on authoritative medical advice and affirming the pivotal role of frontline healthcare workers in pandemic response efforts.

Following the implementation of the COVID-19 opening-up policies, the surge in infections placed immense pressure on medical resources. In this context, healthcare workers continued to work under overwhelming conditions and took on high-risk responsibilities, earning widespread public praise. Keywords such as “gratitude” and “support” strongly reflect the public’s appreciation for the selfless dedication of healthcare personnel. Healthcare workers during public health crises lies not only in their execution of medical techniques but also in providing psychological support and confidence to society. Previous studies have highlighted that healthcare workers, when responding to public health emergencies, endure not only substantial physical labor but also significant psychological stress (71, 72). Therefore, public recognition and appreciation of their work provide not only emotional support but also a vital source of motivation for healthcare workers to continue fulfilling their responsibilities.

In addition, discussions about healthcare workers on Weibo also reflect an increased public trust in the overall healthcare system. The frequent mentions of authoritative experts such as “Zhang Wenhong” highlight the critical role of authoritative medical voices in crisis management. Public recognition and trust in the healthcare system are closely tied to their support for epidemic prevention policies. The credibility of the healthcare system plays a decisive role in the successful implementation of public health policies (73). The high recognition of healthcare workers’ professional abilities reflected in Weibo comments indicates a positive public perception of the overall performance of medical institutions, which has a constructive impact on the advancement of the COVID-19 opening-up policies.

Despite the public’s strong support and gratitude toward healthcare workers, Weibo comments also convey expectations for improved medical resource allocation and long-term reforms. For instance, some users mentioned the hope to increase healthcare resources to optimize the doctor-patient ratio. This indicates that while expressing appreciation for healthcare workers, the public is also placing higher demands on the long-term improvement of the healthcare system. These sentiments not only reflect the public’s support for healthcare workers but also their earnest expectations for further enhancement of the healthcare system.

#### 5.2.2 Trust and support for national policies

Weibo data reveals that keywords like “National Health Commission,” “China,” “Ministry of Foreign Affairs,” “people,” and “epidemic prevention” frequently appear, reflecting strong public recognition of the scientific basis of pandemic prevention policies. High-frequency words such as “policy,” “experts,” and “health” highlight public trust in policies guided by authoritative institutions. Comments such as “I strongly support the timely adjustment of the pandemic prevention policies. I am deeply grateful for the meticulous protection provided by the country over the past 3 years. As we bid farewell to the travel code, my heart is filled with both reluctance and gratitude” reflect the public’s emotional recognition of policy effectiveness and governance care. This trust forms a solid emotional foundation for the successful implementation of policies.

The role of experts is critical, with frequent communication from groups like the “National Health Commission” providing timely scientific guidance and policy explanations. Studies emphasize that the transparency of scientific evidence and the credibility of authoritative institutions are crucial during crises (74). Public recognition of the scientific basis of policies strengthens confidence in the government’s overall governance capabilities. Verbs like “support” and “believe” demonstrate public confidence in achieving policy goals, as evidenced by comments like “The government has promptly introduced optimized policies that have effectively promoted economic recovery. We fully support this.” Such expressions indicate a positive attitude toward policies facilitating economic recovery and reflect trust in the government’s ability to address complex challenges.

China’s collectivist culture emphasizes collective interests, facilitating public trust in national policies. Keywords like “people” and “epidemic prevention” highlight the focus on collective safety. Hofstede (75) noted that collectivist cultures enhance trust in policies, especially during crises, as individuals tend to support measures benefiting the collective. China’s governance model, characterized by efficient execution and adaptability, has deepened public recognition of governance capabilities during the pandemic. Lee et al. (76) found that effective execution and resource allocation are key to building trust. The government’s policy adjustments have supported economic recovery, earning public approval. As one user noted: “The policy adjustments were timely and effective, promoting economic recovery. We fully support this.”

Public trust and support for national policies provide a critical foundation for social resilience in the pandemic’s later stages. Van Bavel et al. (77) emphasized that policy trust enhances social stability and policy effectiveness. Public expressions of support and gratitude on Weibo reflect recognition of policy outcomes and societal confidence in recovery. Through scientific guidance and timely adjustments, the government transformed societal emotions into policy support and optimism for the future. Trust in scientific decision-making, transparent execution, and cultural values collectively underpin public support, providing a strong foundation for policy implementation and recovery.

### 5.3 Acceptance: optimism for normalcy and recovery

With the implementation of COVID-19 opening-up policies, the public has shown increasing acceptance of the new normal, accompanied by optimism about societal recovery. Analysis of Weibo data reveals high-frequency keywords such as “peace,” “happiness,” “recovery,” “safety,” and “good news,” reflecting strong public anticipation for normal life and social security. By sharing hopes for the future, such as, “My heart is full of hope, eagerly anticipating the cancelation of the health code. I wish for the swift return of a society filled with health, happiness, and freedom,” netizens express rising confidence in restoring social order as policies are implemented.

Lighthearted emotional expressions like “haha (laughter)” signal the release of psychological pressure from the pandemic. This shift from anxiety to optimism represents a broader societal transformation. Fredrickson (78) emphasized that positive emotions expand cognitive horizons and enhance resilience, fostering collective adaptability. Through optimism shared on Weibo, the public shows acceptance of life normalizing and trust in policy adjustments. As one user commented: “I firmly believe that every pandemic prevention policy formulated by the national expert team is made with thorough consideration. I hope the public can view these policies rationally, work together to maintain social stability, and avoid being misled by misinformation.” Such sentiments highlight trust in the government’s decision-making and policy transparency.

Van Bavel et al. (77) noted that transparency and scientific decisions enhance public trust, supporting policy acceptance. Keywords like “recovery” and “safety” on Weibo reveal recognition of the scientific basis of opening-up policies and belief in their role in societal recovery. This trust fosters optimism, strengthening confidence in the future and signaling a psychological shift from crisis to recovery. Tugade & Fredrickson (79) observed that positive emotions help individuals and groups adapt to change and prepare for the future, as seen in Weibo discussions where optimism drives public acceptance of policies.

The spread of optimism reflects societal resilience and promotes stability. Trust in policies and optimistic views of life normalization form a shared emotional identity. This collective optimism helps individuals overcome pandemic-induced trauma and supports comprehensive recovery. The tangible benefits of policy adjustments, such as removing health codes, further societal acceptance of the new normal. Studies show that responsive policies enhance trust and acceptance (74). Keywords like “good news” demonstrate recognition of policy outcomes and positive expectations for the future, reflecting the interplay of policy implementation and public emotions.

Public acceptance of normalization and optimism about recovery, driven by trust and policy adjustments, are critical for societal resilience. The scientific basis and transparency of policies promote acceptance and emotional recovery, laying a foundation for societal stability and long-term adaptability.

## 6 Implications

This study, by analyzing the emotional responses of Chinese netizens to the COVID-19 opening-up policies, provides a series of important policy and social governance insights across three dimensions: panic, trust, and acceptance.

Firstly, the study highlights the pronounced supply–demand conflicts in the healthcare system during the initial phase of policy implementation. Issues such as medication hoarding and the strain on funeral home resources exposed inequities in resource distribution, particularly the lack of support for rural and grassroots healthcare institutions. To mitigate these challenges, the government needs to optimize the mechanisms for allocating medical resources and increase support for rural and grassroots areas. Additionally, timely disclosure of medical resource supply and demand data can help reduce public anxiety caused by information asymmetry (80, 81). These measures would help alleviate social panic caused by resource shortages and enhance public trust in the policies.

Secondly, the public’s widespread concern about COVID-19 sequelae reflects a need for long-term medical support and psychological assistance. Sequelae not only have multi-system impacts on physical health but may also lead to mental health issues such as anxiety and depression (82). Therefore, it is recommended that the government and medical institutions increase multidisciplinary research on sequelae to provide a scientific basis for developing rehabilitation strategies. Additionally, more medical resources should be allocated to meet the long-term treatment needs of sequelae patients. Furthermore, authorities should disseminate scientific information about sequelae in a timely manner to alleviate excessive public panic caused by uncertainty (83). Furthermore, a lack of transparency can lead to public doubts about the scientific validity and fairness of policies, potentially triggering social instability. Therefore, the government needs to improve information disclosure mechanisms to ensure clarity and transparency regarding policy rationale, implementation progress, and outcomes. Additionally, enhancing policy communication and public engagement can help the public better understand the goals and scientific basis of policies, thereby increasing societal acceptance.

The study also found that public gratitude and trust toward healthcare workers played a positive role during the pandemic. This sentiment not only served as an important emotional support for policy implementation but also strengthened public trust in the healthcare system. The government can further consolidate this emotional support by highlighting the contributions of healthcare workers. Additionally, organizing interactive events between healthcare workers and the public, such as Q&A sessions or community health education initiatives, can help bridge the gap between government policies and the public.

Finally, the public’s optimistic attitude toward the return to normal life and economic recovery reflects positive emotions during the implementation of the opening-up policies. The spread of optimism can alleviate social pressure and help the public adapt more quickly to policy changes (6). Therefore, it is recommended that the government leverage social media and official communication platforms to share positive cases and scientific explanations, encouraging the public to approach policy adjustments with rationality and optimism. Additionally, supporting community-level mutual aid activities can foster a positive social atmosphere, further promoting public acceptance of the policies.

In summary, this study provides insights into optimizing policies and social governance from three dimensions: panic, trust, and acceptance. The recommendations include improving the allocation of medical resources, advancing the development of post-sequelae support systems, enhancing policy transparency, strengthening the public image of healthcare workers, and promoting the spread of optimism. These suggestions not only offer valuable references for policymaking in future public health crises but also lay a theoretical and practical foundation for maintaining social stability and public trust.

## 7 Conclusion, contributions and limitations

This study, by analyzing Chinese netizens’ emotional responses to the COVID-19 opening-up policies, reveals the complex dynamics of public emotions during the early stages of policy implementation. These emotions are primarily manifested in three dimensions: panic, trust, and acceptance. Panic centered on the shortage of medical resources, concerns over COVID-19 sequelae, and doubts about policy transparency and fairness; trust was reflected in public gratitude toward healthcare workers and support for national policies; and acceptance demonstrated the public’s optimistic attitude toward returning to normal life. The study indicates that changes in public emotions not only reflect the societal impact of policy implementation but also highlight the critical roles of medical resource allocation, information transparency, and psychological support in the adjustment of pandemic policies.

This study applies sentiment analysis methods to social media data, offering a new perspective for understanding public emotional dynamics during significant public health policy changes. Specifically, the focus on emotional responses within China’s unique socio-cultural context addresses gaps in existing literature on sentiment studies in the context of opening-up policies. Furthermore, this research enriches the theoretical framework in the fields of crisis management and policy communication, uncovering the interactive roles of panic, trust, and acceptance in policy implementation.

This study provides valuable practical guidance for public policymakers on managing public emotions. By highlighting the significant impact of medical resource allocation and information transparency on public sentiment, it recommends that governments prioritize the equitable distribution of medical resources, transparency in policy communication, and addressing public concerns about long-term sequelae. Additionally, the study emphasizes the critical role of social media as a tool for guiding public opinion, offering practical insights for developing effective social communication strategies during public health crises.

Although this study provides important insights into the dynamics of public emotions, it has the following limitations: First, this study focuses on the context of China, and the emotional responses observed may differ significantly from those in other countries and regions during pandemic policy adjustments, limiting the international applicability of the findings. Future research could expand the scope to include other countries and regions, comparing public emotional responses to similar policies across different cultural contexts to explore how cultural differences influence emotional expression and policy acceptance. Second, the data analyzed in this study are concentrated on the early stages of the implementation of COVID-19 opening-up policies, failing to fully capture the changes in public emotions over the longer term. This may result in conclusions that predominantly reflect initial emotional reactions, overlooking potential shifts in sentiment over time. Future research could employ longitudinal data analysis to more comprehensively reveal the long-term trends in emotional dynamics. Additionally, the lexicon-based approach we used is relatively simple, future research will explore more sophisticated methods, including deep learning and machine learning, to improve the precision and complexity of sentiment analysis.

## Data Availability

The original contributions presented in the study are included in the article/supplementary material, further inquiries can be directed to the corresponding author.

## Footnotes

^1^ https://open.weibo.com/
